# Impact of Short-Term Exposure to Non-Functionalized Polystyrene Nanoparticles on DNA Methylation and Gene Expression in Human Peripheral Blood Mononuclear Cells

**DOI:** 10.3390/ijms252312786

**Published:** 2024-11-28

**Authors:** Kinga Malinowska, Kateryna Tarhonska, Marek Foksiński, Paulina Sicińska, Ewa Jabłońska, Edyta Reszka, Ewelina Zarakowska, Daniel Gackowski, Karolina Górecka, Aneta Balcerczyk, Bożena Bukowska

**Affiliations:** 1 Department of Biophysics of Environmental Pollution, Faculty of Biology and Environmental Protection, University of Lodz, Pomorska Str. 141/143, 90-236 Lodz, Poland; kinga.malinowska@edu.uni.lodz.pl (K.M.); paulina.sicinska@biol.uni.lodz.pl (P.S.); edyta.reszka@biol.uni.lodz.pl (E.R.); 2 Department of Translational Research, Nofer Institute of Occupational Medicine, Teresy Str. 8, 91-348 Lodz, Poland; kateryna.tarhonska@imp.lodz.pl (K.T.); ewa.jablonska@imp.lodz.pl (E.J.); 3 Department of Clinical Biochemistry, Faculty of Pharmacy, Collegium Medicum in Bydgoszcz, Nicolaus Copernicus University in Toruń, 85-092 Bydgoszcz, Poland; marekf@cm.umk.pl (M.F.); ewelinaz@cm.umk.pl (E.Z.); danielg@cm.umk.pl (D.G.); 4 The Bio-Med-Chem Doctoral School, University of Lodz, 90-237 Lodz, Poland; karolina.gorecka@edu.uni.lodz.pl; 5 Lodz Institutes of the Polish Academy of Sciences, University of Lodz, 90-237 Lodz, Poland; 6 Department of Oncobiology and Epigenetics, Faculty of Biology and Environmental Protection, University of Lodz, Pomorska 141/143, 90-236 Lodz, Poland; aneta.balcerczyk@biol.uni.lodz.pl

**Keywords:** polystyrene nanoparticles, peripheral blood mononuclear cells, epigenetic DNA modifications, suppressor genes, proto-oncogenes

## Abstract

The aim of the present study was to investigate the concentration- and size-dependent effects of non-functionalized polystyrene nanoparticles (PS-NPs) of varying diameters (29 nm, 44 nm, and 72 nm) on specific epigenetic modifications and gene expression profiles related to carcinogenesis in human peripheral blood mononuclear cells (PBMCs) in vitro. This in vitro human-cell-based model is used to investigate the epigenetic effect of various environmental xenobiotics. PBMCs were exposed to PS-NPs at concentrations ranging from 0.001 to 100 µg/mL for 24 h period. The analysis encompassed epigenetic DNA modifications, including levels of 5-methyl-2′-deoxycytidine (5-mdC) and 5-(hydroxymethyl)-2′-deoxycytidine (5-hmdC), as well as the levels of 2′-deoxyuridine (dU) and 5-(hydroxymethyl)-2′-deoxyuridine (5-hmdU) by mass spectrometry methods, methylation in the promoter regions of selected tumor suppressor genes *TP53* (P53), *CDKN2A* (P16), and *CDKN1A* (P21) and proto-oncogenes (*CCND1*, *BCL2*, *BCL6*), along with the expression profile of the indicated genes by real-time PCR assays. The results obtained revealed no significant changes in global DNA methylation/demethylation levels in PBMCs after short-term exposure to non-functionalized PS-NPs. Furthermore, there were no changes observed in the level of dU, a product of cytosine deamination. However, the level of 5-hmdU, a product of both 5-hmdC deamination and thymine oxidation, was increased at the highest concentrations of larger PS-NPs (72 nm). None of the PS-NPs caused a change in the methylation pattern of the promoter regions of the *TP53*, *CDKN2A*, *CDKN1A*, *CCND1*, *BCL2* and *BCL6* genes. However, gene profiling indicated that PS-NPs with a diameter of 29 nm and 44 nm altered the expression of the *TP53* gene. The smallest PS-NPs with a diameter of 29 nm increased the expression of the *TP53* gene at a concentration of 10 µg/mL, while PS-NPs with a diameter of 44 nm did so at a concentration of 100 µg/mL. An increase in the expression of the *CDKN2A* gene was also observed when PBMCs were exposed to PS-NPs with 29 nm in diameter at the highest concentration. The observed effect depended on both the concentration and the size of the PS-NPs.

## 1. Introduction

Plastic, a versatile material with myriad applications, has gradually become a global environmental concern due to the ubiquity of its residues and waste. According to the OECD report, global plastic consumption is projected to surge from 460 million tons (Mt) in 2019 to 1231 Mt in 2060. Regrettably, two-thirds of this consumption comprises single-use products that quickly become waste, contaminating soil, rivers, and oceans [1]. Poor waste management practices, landfilling, inappropriate disposal, or runoff from industrial and agricultural activities are the primary sources of environmental plastic pollution [2,3]. Micro- and nanoplastics (MNPLs), derived from macroplastics, are termed secondary particles, however, MNPLs can also come from primary sources, intentionally produced for commercial applications, collectively constituting global pollutants [4,5].

Various classifications of plastic particles exist. One classification defines microplastics (MPs) as particles ranging from 1 μm and 1 mm in size and nanoplastics (NPs) as particles smaller than 1 μm (ISO/TR 21960:2020) [6]. In Europe, the nanoscale definition was provided by the International Organization for Standardization (ISO) in cooperation with the European Committee for Standardization (CEN), defining it as a size range from approximately 1 to 100 nm [7].

Human exposure to MNPLs occurs through animal- and plant-based, food additives, beverages, and plastic food packaging [8]. Due to their small size, MNPLs can be absorbed by plants, ingested by animals, and consequently accumulate in the food chain [5,9], thereby entering the bodies of animals and humans [10,11,12]. Studies on *Caenorhabditis elegans* have shown that nanoplastics at environmental concentrations can cause transgenerational changes in germline ced-1 expression [13].

In vitro and in vivo studies have shown that NPs possess the capability to cross biological barriers [14,15,16], including the blood–brain barrier [17,18,19]. Consequently, plastic particles accumulating in cells and tissues can induce biological effects and pose a potential threat to human health [20]. The potential carcinogenic effects of micro- and nanoplastics are under investigation. Initial reports in this area have indicated the induction of ovarian cancer in mice by PS-NPs [21] and the induction of liver cancer by polyvinyl chloride microplastic [22,23]. Other reports have highlighted a higher number of MPs in the tumor tissue of patients with colorectal adenocarcinoma [23,24]. Plastic particles have been shown to disrupt the colonic mucus layer, thus reducing its protective function, and increasing the likelihood of colorectal cancer [21,25]. Additionally, the study of Brynzak-Schreiber et al. [26], underscored the potential of MNPs as hidden catalysts for tumor progression, particularly through enhancing cell migration and possibly fueling metastasis. Researchers demonstrated the persistence and bioaccumulation of MNPLs in colorectal cancer cell lines, indicating crucial toxicological traits of MNPLs as substances of concern under the European Regulation of REACH (Regulation concerning the Registration, Evaluation, Authorisation and Restriction of Chemicals). NPs may cause exacerbated inflammatory damage in animals with intestinal immune imbalance [27,28] and alter the gut microbiota in their bodies [29].

MNPLs have the potential to induce DNA damage in human cells, as demonstrated in the in vitro studies [30,31,32] and cause epigenetic modifications. Epigenetic modifications resulting from environmental pollutants contribute to numerous human diseases, including cancer [33,34]. To date, there are no data on epigenetic changes (e.g., total DNA methylation) under the influence of plastic NPs in humans.

The methylation of cytosine (C), a pivotal epigenetic DNA modification, is closely associated with gene repression, profoundly influencing cellular identity. Active DNA demethylation, on the other hand, leads to the activation of previously silenced genes. The molecular mechanism of active DNA demethylation involves TET (ten-eleven translocation) proteins that catalyze the oxidation of 5-methylcytosine (5-mC) to 5-hydroxymethylcytosine (5-hmC), and subsequently to 5-formylcytosine (5-fC), which eventually converts to 5-carboxycytosine (5-caC). Experimental studies have demonstrated that TETs are also involved in the synthesis of 5-hydroxymethyluracil (5-hmU), a compound with epigenetic function. Additionally, it is also possible that 5-hmC and C may also undergo deamination by activation-induced cytidine deaminase AID/APOBEC-family, generating 5-hydroxymethyluracil and uracil, respectively [35,36].

The methylation of promoter regions suppressor genes such as *CDKN2A* encoding p16 protein, *TP53*–p53 protein and *CDKN1A*–p21 protein, along with proto-oncogenes *CCND1*, *BCL2*, *BCL6* and their subsequent expression determine the appropriate levels of individual proteins essential for proper cell functioning. Disturbances in their expression may cause changes in the cell cycle, induction of apoptosis, or alterations in the cellular aging process. Proteins like p16 and p53 are crucial components of two major cell cycle control pathways frequently implicated in human tumorigenesis. Dysregulation of the p16 or p53 pathways is observed in the vast majority of human cancers [37,38,39]. The products encoded by the *TP53/CDKN2A*/*CDKN1A* genes influence DNA damage repair pathways, which are commonly impaired in human cancers [40]. Additionally, research suggests that p53 protein, acting as a transcription factor, regulates numerous vital cellular pathways, including apoptosis, and may contribute to neuronal death characteristic neurodegenerative diseases [41].

In this context, we employed our in vitro model of human peripheral blood mononuclear cells (PBMCs) of three non-functionalized PS-NPs with varying diameters 29 nm, 44 nm and 72 nm to contribute new data and enhance understanding of the potential health risks associated with plastic particles. In this context, we applied our in vitro model of human peripheral blood mononuclear cells (PBMCs) with three non-functionalized PS-NPs with varying diameters 29 nm, 44 nm and 72 nm to contribute new data and to enhance understanding of the potential health risks associated with plastic particles. PBMCs are isolated from peripheral blood and identified as any blood cell with a round nucleus (i.e., lymphocytes, monocytes or natural killer cells (NK cells)). These cells are commonly used to investigate the toxic effect of different xenobiotics [42,43,44], especially on their epigenetic parameters, e.g., glyphosate [45,46], its metabolite AMPA [47] and phthalates [48].

The potential effect of PS-NPs on the molecular markers and determinants of the carcinogenesis process was investigated. The cytotoxicity of PS-NPs was analyzed in our previous studies [31,49]. Based on those findings, we selected concentrations for the current study ranging from 0.001 to 100 µg/mL. Within this range, PS-NPs showed no cytotoxicity. We evaluated the impact of PS-NPs on selected markers of epigenetic regulation, including DNA modifications, i.e., levels of 5-methyl-2′-deoxycytidine (5-mdC) and 5-(hydroxymethyl)-2′-deoxycytidine (5-hmdC) as well as level of 2′-deoxyuridine level (dU) and 5-(hydroxymethyl)-2′-deoxyuridine (5-hmdU). Furthermore, we examined the methylation status of promoter regions of selected tumor suppressor genes (*CDKN2A*, *TP53* and *CDKN1A*) and proto-oncogenes (*CCND1*, *BCL2*, *BCL6*), alongside investigating their expression levels.

## 2. Results

### 2.1. Effect of PS-NPs on DNA Methylation Determinants (5-mdC, 5-hmdC, dU and 5-hmdU) in Peripheral Blood Mononuclear Cells

**Figure 1:**
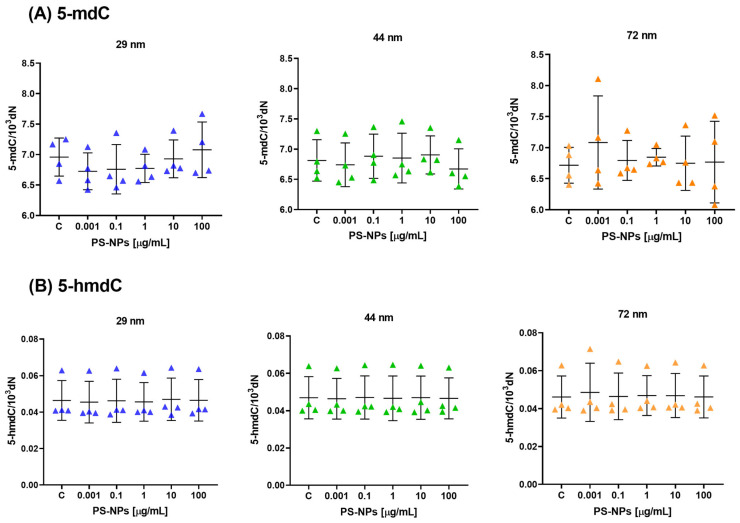
The level of (**A**) 5-mdC and (**B**) 5-hmdC per 10^3^ dN (10^3^ deoxyribonucleotides) in human PBMCs incubated with PS-NPs of 29, 44, and 72 nm in diameter (0.001–100 μg/mL) for 24 h period. The data are presented as mean ± SD, n = 4 independent experiments. Statistical analysis was conducted using one-way ANOVA or Kruskal–Wallis test.

The analysis of DNA methylation determinants showed that there were no statistically significant changes observed in the levels of 5-mdC and 5-hmdC in peripheral blood mononuclear cells (PBMCs) when treated with PS-NPs of various diameters (29 nm, 44 nm, and 72 nm) at different concentrations (ranging from 0.001 to 100 μg/mL) for a duration of 24 h (Figure 1A,B). There were no statistically significant differences between the individual concentrations and the control group, as assessed using either the ANOVA or Kruskal–Wallis test. The study conducted four individual experiments to gather data, and the mean ± standard deviation (SD) was calculated to analyze the results.

**Figure 2:**
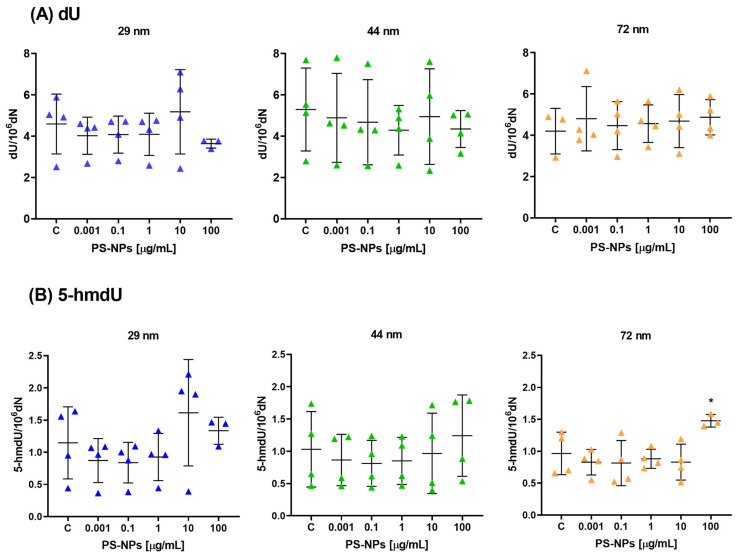
The level of (**A**) dU and (**B**) 5-hmdU per 10^6^ dN (10^6^ deoxyribonucleotides) in human PBMCs incubated with PS-NPs of 29, 44, and 72 nm in diameter (0.001–100 μg/mL) for 24 h period. The data are presented as mean ± SD, n = 4 independent experiments. Statistical analysis was conducted using one-way ANOVA or Kruskal–Wallis test, followed by relevant post hoc; * *p* < 0.05.

Also, the results indicate that there were no statistically significant differences observed in the level of 2′-deoxyuridine (dU) when analyzing the impact of PS-NPs across various concentrations (Figure 2A). However, the study did find statistically significant differences in the level of 5-(hydroxymethyl)-2′-deoxyuridine (5-hmdU) specifically at the highest tested concentration of NPs, which was 100 µg/mL, for the diameter of 72 nm (Figure 2B). This suggests that while dU levels remained unaffected by the PS-NP treatment, the presence of NPs, particularly at the highest concentration and specific diameter, influenced the level of 5-hmdU.

### 2.2. Analysis of Methylation at Promoter Regions of the Selected Tumor Suppressor Genes

**Figure 3:**
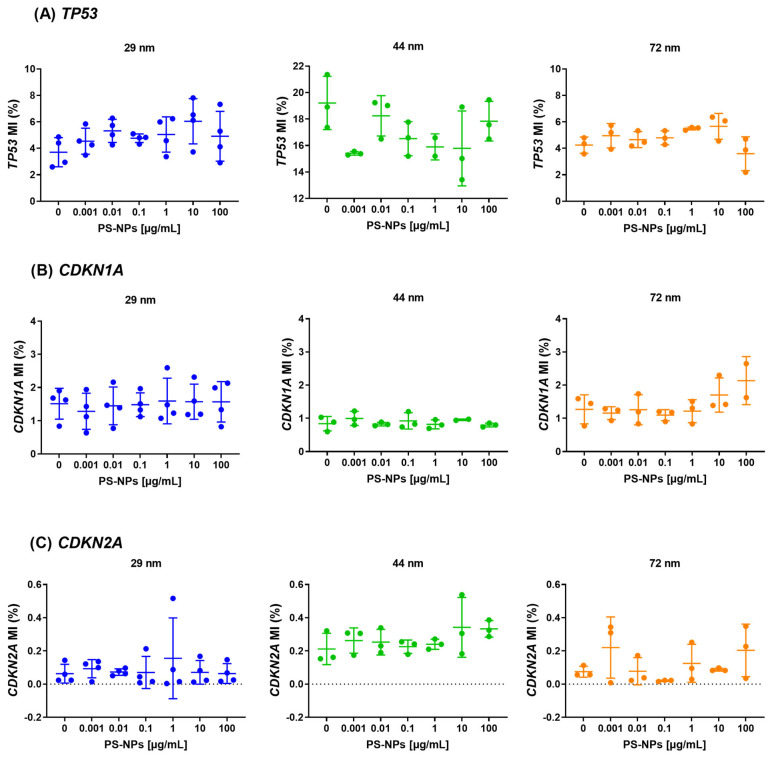
Methylation of suppressor genes (*TP53*, *CDKN2A*, *CDKN1A*) in human PBMCs incubated with PS-NPs of 29, 44, and 72 nm in diameter (0.001–100 μg/mL) for 24 h period. The data are presented as mean ± SD, n = 3 to 4 independent experiments. The promoter methylation experiments for 29, 44, and 72 nm were performed on separate plates, each including a respective control group (cells incubated in medium only). Consequently, baseline MI levels varied for some genes across different PS-NP sizes. Statistical analysis was conducted using one-way ANOVA or Kruskal–Wallis test.

The analysis of methylation in the promoter regions of the tested genes revealed no statistically significant changes attributed to exposure to PS-NPs across various diameters (29 nm, 44 nm, and 72 nm) and concentrations ranging from 0.001 to 100 µg/mL. Specifically, no statistically significant alterations in methylation were observed in the promoter regions of the selected tumor suppressor genes, including *CDKN2A*, *TP53*, and *CDKN1A* (Figure 3). The significance level was set at *p* < 0.05.

### 2.3. Analysis of Methylation at Promoter Regions of the Selected Proto-Oncogenes

**Figure 4:**
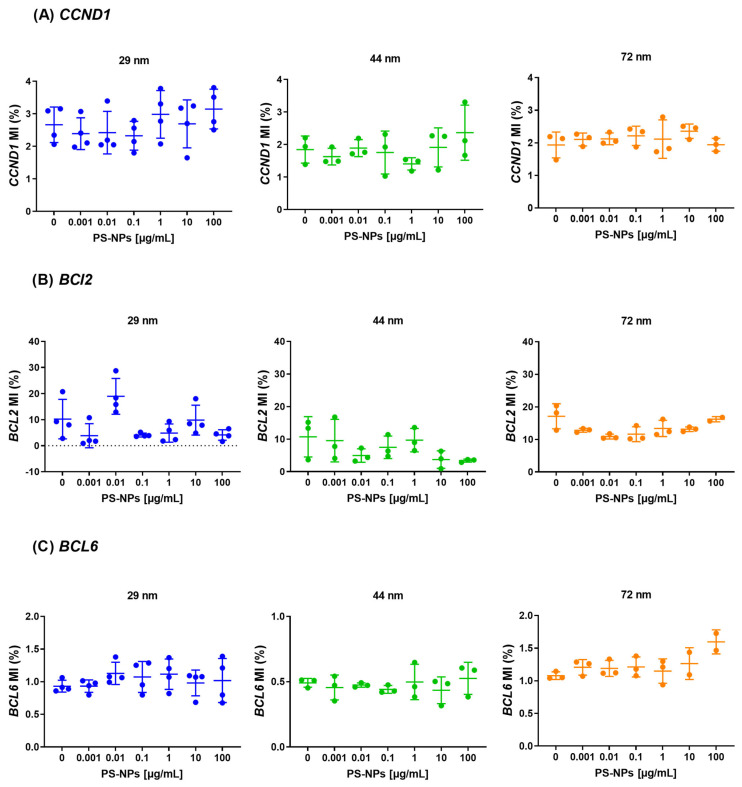
Methylation of proto-oncogenes (*CCND1*, *BCL2*, *BCL6*) in human PBMCs incubated with PS-NPs of 29, 44, and 72 nm in diameter (0.001–100 μg/mL) for 24 h period. The data are presented as mean ± SD, n = 3 to 4 independent experiments. The promoter methylation experiments for 29, 44, and 72 nm were performed on separate plates, each including a respective control group (cells incubated in medium only). Consequently, baseline MI levels varied for some genes across different PS-NP sizes. Statistical analysis was conducted using one-way ANOVA or Kruskal–Wallis test.

The investigation did not detect statistically significant (*p* < 0.05) alterations in methylation levels at the promoter regions of the selected proto-oncogenes, including *CCND1*, *BCL2*, and *BCL6* (Figure 4). This implies that 24 h exposure to various diameters of PS-NPs and tested concentrations, did not affect the methylation pattern in the promoter regions of analyzed proto-oncogenes in PBMCs.

### 2.4. Analysis of Gene Expression of the Selected Tumor Suppressor Genes

**Figure 5:**
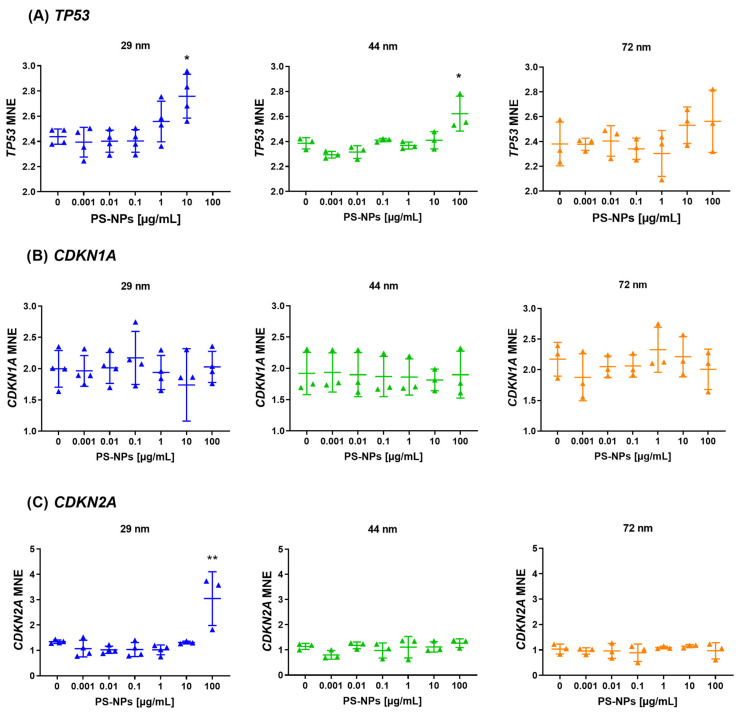
Expression of suppressor genes (*TP53*, *CDKN2A* and *CDKN1A*) in human PBMCs incubated with PS-NPs of 29, 44, and 72 nm in diameter (0.001–100 μg/mL) for 24 h period. The data are presented as mean ± SD, n = 3 to 4 independent experiments. Statistical analysis was conducted using one-way ANOVA or Kruskal–Wallis test, followed by relevant post hoc test, * *p* < 0.05, ** *p* < 0.001.

The study identified a statistically significant increase in *TP53* gene expression in response to exposure to PS-NPs with diameters of 29 nm and 44 nm at the highest analyzed concentrations. Specifically, a significant increase in *TP53* gene expression was observed at 10 µg/mL for 29 nm PS-NPs (*p* = 0.017, compared to control) and at 100 µg/mL for 44 nm PS-NPs (*p* = 0.008, compared to control). For the *TP53* gene after incubation PBMCs with PS-NPs of 29 nm in diameter, the expression analysis did not include the highest concentration (100 µg/mL) due to technical issues (the absence of fluorescence signal during qPCR for two from four batches of samples). Additionally, an increase in expression was noted for the *CDKN2A* gene when exposed to 29 nm PS-NPs at the highest concentration of 100 µg/mL (*p* = 0.0009, compared to control) (Figure 5).

However, there were no changes observed in the expression of the *CDKN1A* gene following incubation with PS-NPs with a diameter of 29 nm. Furthermore, exposure to PS-NPs with a diameter of 72 nm did not impact the expression of the tested genes (Figure 5).

### 2.5. Analysis of Gene Expression of the Selected Proto-Oncogenes

**Figure 6:**
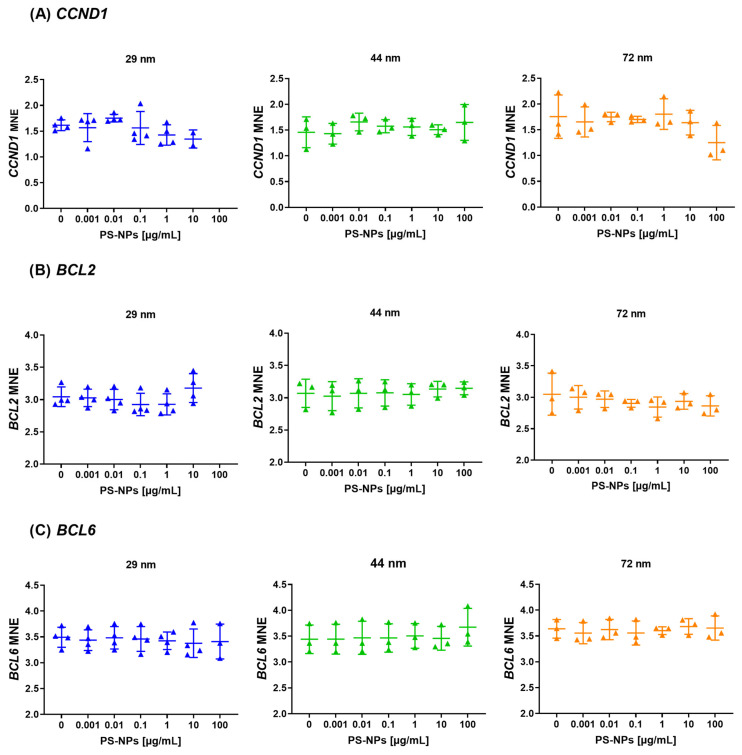
Expression of proto-oncogenes (*CCND1*, *BCL2*, *BCL6*) in human PBMCs incubated with PS-NPs of 29, 44, and 72 nm in diameter (0.001–100 μg/mL) for 24 h period. The data are presented as mean ± SD, n = 3 to 4 independent experiments. Statistical analysis was conducted using one-way ANOVA or Kruskal–Wallis test.

The performed analysis found no significant changes in the expression levels of proto-oncogenes, including *CCND1*, *BCL2* and *BCL6*, following incubation with all PS-NPs (Figure 6). For 2 genes (*CCND1*, *BCL2*) after incubation PBMCs with PS-NPs of 29 nm in diameter, the expression analysis did not include the highest concentration (100 µg/mL) due to technical issues (the absence of fluorescence signal during qPCR for three from four batch of samples). This suggests that exposure to PS-NPs across various diameters and concentrations did not have a discernible impact on the expression of these proto-oncogenes under the experimental conditions examined.

## 3. Discussion

Epigenetic alterations in living organisms might be influenced by a plethora of environmental factors, also including specific types of pollutants with widely used herbicides. It was identified that both glyphosate [45,46], its metabolite AMPA [50] and phthalates [48], may affect total DNA methylation and methylation in the promoter regions of several tumor suppressor genes and proto-oncogenes in human PBMCs. Such alterations were observed even at short-term 24 h exposure of cells. Recently, MNPLs have also gained a lot of attention, due to their tendency to accumulate in the food chain of animals, which creates a risk to humans. Furthermore, MNPLs can enter human organisms not only through the gastrointestinal tract but also through pulmonary and skin exposure [51]. However, data on the effects of plastic nanoparticles on the epigenome are relatively scarce. As it was demonstrated, exposure to PS-NPs (100 nm) caused elevation in mir-38 levels (germline miRNA) in the model organism *Caenorhabditis elegans* [52]. Moreover, other studies have shown that combined exposure of marine organisms, copepods to ocean acidification and nanoparticles was associated with altered methylation of specific genes, which resulted in reproduction impairments [53]. In turn, Stojkovic et al. found that exposure to polystyrene (PS) nanoplastic particles alters the transcriptomic and epigenomic profiles of human fibroblasts and induced pluripotent stem cells (hiPSCs), affecting genes and pathways related to pluripotency, cancer, inflammation, metabolism, and immunity. The findings highlight the potential of hiPSC models to identify molecular biomarkers of environmental pollution and provide insights into the origins of environmental diseases [54].

Nevertheless, the impact of PS-NPs on human epigenome from the level of disruption in DNA methylation patterns or changes within miRNA signaling remains poorly understood.

In our previous study, we investigated the role of PS-NPs in PBMCs and it was observed adverse effects of nanoparticles, including induction of both single and double-stranded DNA breaks as well as purine and pyrimidine oxidation under treatment with PS-NPs of various sizes and concentrations [31]. Additionally, our findings showed the possibility of initiating apoptosis in the PMBCs model by PS-NP treatment [55], which was consistent with the results obtained by Li et al., [56]. They observed the upregulated expression of several apoptotic-related proteins in murine splenic lymphocytes by nanoplastic particles. The effects of PS-NPs on DNA identified so far, along with literature findings showing that DNA damage and repair can lead to an accumulation of aberrant DNA methylation patterns and contribute to the epigenetic control of gene expression [57], prompted us to examine the impact of plastic NPs on changes in methylation status in the promoters of genes crucial to cell cycle/cell death control, including *TP53*, *CDKN2A* or *BCL2*.

This study aimed to investigate the influence of non-functionalized PS-NPs of varying diameters (29 nm, 44 nm, and 72 nm) on selected epigenetic parameters in human peripheral blood mononuclear cells (PBMCs) in vitro, over a short-term exposure of 24 h.

Our findings revealed no significant changes in global DNA methylation/demethylation levels in PBMCs exposed to PS-NPs, as indicated by unchanged levels of 5-methylcytosine and 5-hydroxymethylcytosine. This is consistent with several studies indicating that while nanoparticles can enter cells and potentially cause genotoxicity, not all nanoparticle exposures result in global epigenetic modifications. For instance, a study by Brzoska et al. (2019) demonstrated that certain types of nanoparticles, such as silver (AgNPs), gold (AuNPs), and superparamagnetic iron oxide (SPIONs) with a particle size of 20 nm did not alter global DNA methylation patterns in human HepG2 cell line [58]. Interestingly, our study found an increase in the level of 5-hmdU, a product of both 5-hmdC deamination and thymine oxidation, which as identified in mouse embryonic stem cells, may affect protein-DNA interactions and transcription factor binding. As presented, 5-hmdU as a base recruits transcription factors and proteins that are involved in chromatin biochemistry, including Uhrf1 and Uhrf2 [59]. In our experimental conditions, we found increased level of 5-hmdU, due to treatment with the highest concentration (100 μg/mL) of larger PS-NPs (72 nm). The precise causes regarding elevated levels of 5-hmdU in our study have not been unveiled, thus requiring further investigation. However, we hypothesize that they may be the consequence of excessive ROS formation upon PS-NP treatment [49]. Nevertheless, [59] demonstrated that a relatively low amount of 5-hmdU is generated through 5-hmC deamination or reactive oxygen species, while the majority of 5-hmU is generated from thymine oxidation by TET enzymes in mouse embryonic stem cells. 5-hmU formation upon oxidation of thymine may also explain the lack of changes in 5-mdC and 5-hmdC in our study.

Epigenetic modifications, including DNA methylation, are critical for gene regulation. Environmental pollutants, such as benzene, persistent organic pollutants, metals, and air pollution, have been shown to induce epigenetic changes, leading to many different health outcomes [60]. Epidemiologic studies have reported associations between global white blood cell methylation and several different cancers, including cancers of the colon, bladder, colorectal neoplasms, stomach, breast and head and neck [61,62,63]. In our experimental model, no changes were observed in the methylation patterns of the promoter regions of tumor suppressor genes (*TP53*, *CDKN2A*, *CDKN1A*) and proto-oncogenes (*CCND1*, *BCL2*, *BCL6*), indicating that PS-NPs might not directly affect gene-specific methylation under the experimental conditions, but also we should have in mind the experimental conditions, particularly short-term 24 h exposure. However, gene expression analysis revealed that PS-NPs with diameters of 29 nm and 44 nm significantly altered the expression of the *TP53* gene, a gene that plays a key role in cancer prevention, and at the same time, is the most frequently mutated gene (>50%) in human cancers [64]. Specifically, 29 nm PS-NPs increased *TP53* expression at a concentration of 10 µg/mL, while 44 nm PS-NPs did so at 100 µg/mL. Additionally, an increase in *CDKN2A* gene expression was observed with 29 nm PS-NPs at the highest concentration (100 µg/mL). These findings underscore the sensitivity of gene expression to nanoparticle size and concentration, echoing results from other studies where nanoparticle exposure led to size-dependent variations in gene expression profiles. Numerous studies tend to attribute the effects mediated by nanoparticles to their sizes, pointing to the possibility that smaller NPs are more likely to enter and accumulate within various cells and tissues and thereby affect their physiological activity [65,66,67,68], which was also the case in our study. However, recent studies performed on mouse skin cells also show that high diameter particles 200–6000 nm alter gene expression and increase the expression of the tumor suppressor protein p53 after 24 h exposure [69].

Data regarding the impact of plastic nanoparticles on the regulation of gene expression sometimes remain conflicting, mainly due to variations in nanoparticle types, sizes, concentrations, experimental conditions, time of exposure, and the tendency of NPs to form local agglomerates. Nevertheless, so far, the ongoing conclusion is definitely that PS-NPs have an impact on gene expression, also involved in the regulation of the cell cycle and apoptosis process [70,71]. Forte et al. demonstrated the differential expression of cell cycle-associated genes, including *c-Myc*, *ERK-1*, *Ki67*, *CCNE1*, and *P38*, in human gastric adenocarcinoma cells incubated with PS-NPs of 44 nm and 100 nm in diameter [67]. Moreover, in mouse spermatogonia-derived GC-1 treated with PS-NPs with a diameter of 80 nm, an increase in the expression genes involved in the life/dead machinery, which means *CDKN2A*, *CDKN1A*, and *RB1* were detected measured after 120 h of exposure [72]. Martin-Folgar et al. observed an increase in the expression of anti-apoptotic *BCL2* [71], whereas Gonzalez-Caballero et al. reported the opposite [18], despite using the same cellular model (human neuronal stem cells) and relatively similar diameters of nanoparticles (30 nm and 25 nm, respectively). In our studies on PBMCs, we did not observe changes in *BCL2* expression at any concentration or for any of the nanoparticle sizes tested: 29 nm, 44 nm, or 72 nm. However, our previous research revealed that PS-NP exposure triggers apoptosis in PBMCs by activating either the intrinsic or extrinsic pathway in a size-dependent manner [55].

Plastic nanoparticles are ubiquitous in the environment due to widespread plastic production, pollution and bioaccumulation. These particles are not only difficult to degrade and persistent but also have the potential to accumulate in living organisms, raising concerns about their impact on humans. One critical area of concern is health and their potential role in carcinogenesis, which can be mediated through epigenetic modifications and changes in gene expression. Our results suggest that 24 h exposure of PBMCs to PS-NPs does not significantly alter global DNA methylation or the methylation of specific gene promoters; however, it does induce changes in gene expression, particularly for genes like *TP53* and *CDKN2A*. The most pronounced effects were observed with smaller PS-NPs (29 nm), highlighting the importance of nanoparticle size in determining their biological impact. These findings contribute to the growing body of the literature indicating that nanoparticles can modulate cellular processes in a size- and concentration-dependent manner, with potential implications for their safety and regulatory assessment [73,74].

In summary, our results suggest that PS-NPs have a modest potential to influence epigenetic parameters in PBMCs in vitro under short-term exposure of a 24 h period. Among the tested nanoparticle sizes, the most pronounced effects were observed with PS-NPs of 29 nm diameter. This finding underscores the importance of nanoparticle size in determining their epigenetic impact.

A limitation of the study is the short-term exposure model used for PS-NPs, which only approximates the conditions experienced by the human body. This suggests that our research serves as a preliminary step toward broader, population-based analyses. In future studies, to further investigate this issue, it will be essential to assess the effects of nanoparticles on genes directly involved in DNA methylation and demethylation, as well as genes related to chromatin remodeling, including those that regulate histone methylation and histone acetylation status.

## 4. Materials and Methods

### 4.1. Chemicals

Standards of non-functionalized PS-NPs were purchased from Polysciences Europe GmbH (Hirschberg an der Bergstraße, Germany) (29 nm—catalogue number: PS02001; 44 nm—catalogue number: PS02002; 72 nm—catalogue number: PS02003). PBMCs separation medium (Lymphosep) (1.077 g/cm^3^) was sourced from Biowest (Nuaillé, France), RPMI 1640 medium from Gibco (Thermo Fisher Scientific Inc., (Waltham, MA, USA), HBSS solution from Merck (Burlington, MA, USA), and penicillin and streptomycin were obtained from Merck (Burlington, MA, USA).

For laboratory procedures, we utilized the following kits and reagents: PureLink™ Genomic DNA mini kit (Thermo Fisher Scientific, Waltham, MA, USA; catalogue number K182001), First Strand cDNA Synthesis Kit (Thermo Fisher Scientific, Waltham, MA, USA; catalogue number K1612) and FastStart Essential DNA Green Master from Roche (Basel, Switzerland; catalogue number 04896866001). Methyl Primer Express^®^, v.1.0 was obtained from Thermo Fischer Scientific (Waltham, MA, USA). TRIzol™ Plus RNA purification kit was purchased from Thermo Fischer Scientific (Waltham, MA, USA, catalogue number 12183555). CpG methylated Jurkat genomic DNA (catalogue number N4002S) and 5-Azadc-treated Jurkat genomic DNA (catalogue number N4003S) were procured from NEB (Ipswich, MA, USA), and the MethylCode™ Bisulfite Conversation kit was sourced from Thermo Fisher Scientific (Waltham, MA, USA; catalogue number MECOV50).

Chemicals for DNA isolation and tetrahydrouridine were purchased from Merck (Burlington, MA, USA). Nuclease P1 and shrimp alkaline phosphatase were obtained from NEB (Ipswich, MA, USA). Non-labeled genuine 5-methyl-2′-deoxycytidine (5-mdC), 5-(hydroxymethyl)-2′-deoxycytidine (5-hmdC) and 5-(hydroxymethyl)-2′-deoxyuridine (5-hmdU) were purchased from Berry & Associates (LGC Biosearch Technologies, Hoddesdon, UK); 2′-deoxyuridine (dU), 2′-deoxythymidine (dT), 2′-deoxyadenosine (dA), 2′-deoxyguanosine (dG), and 2′-deoxycytidine (dC) were purchased from Merck (Burlington, MA, USA). Stable-isotope-labeled internal standards of [^15^N−U,^13^C−U]-2′-dT were procured from Cambridge Isotope Laboratories (Tewksbury, MA, USA), [^13^C,^15^N_2_]-2′-dU from Medical Isotopes (Pelham, NH, USA), and [D_3_]-5-hmdC from Toronto Research Chemicals Inc. (North York, ON, Canada). [^15^N_2_,^13^C_10_]-5-mdC was synthesized following the method described by Divakar and Reese [75] using [^15^N−U,^13^C−U]-2′-dT as a substrate. Chromatographically purified [^15^N_2_,^13^C_10_]-5-mdC and [^15^N−U,^13^C−U]-2′-deoxythymidine (5 mg) were further oxidized with Na_2_S_2_O_8_ (25 mg/mL in 0.1 M phosphate buffer pH 7.0) to obtain [^15^N_2_,^13^C_10_]-5-hmdU (20 min at 60 °C), respectively, using the optimized method described by Abdel Rahman et al. [76]. Other chemicals were from Carl Roth (Karlsruhe, Germany) and POCh (Gliwice, Poland) and were of analytical grade.

### 4.2. Biological Material

Human peripheral blood mononuclear cells (PBMCs) were isolated from the leukocyte-buffy coat obtained from the Regional Centre for Blood Donation and Treatment (RCBDT) in Lodz, Poland. The acquisition of blood for this research was made possible through a contract between the University of Lodz and the aforementioned RCBDT. The leukocyte buffy coat was isolated by bank personnel from whole blood donated by healthy, non-smoking individuals. The Lodz Blood Bank holds accreditation from the Minister of Health (No. BA/2/2004) and our described experiments received approval from the Bioethics Committee of the University of Lodz (Resolution No. 8/KBBN-UŁ/II/2019 (8 April 2019)). Cell isolation procedures followed the methodology outlined by [77]. The PBMCs’ final density utilized in the experiments (after addition of PS-NP) was 1 × 10^6^ cells/mL. The in vitro PBMCs-based model presented was used in our previous studies examining the genetic [45,78,79] and epigenetic effects of glyphosate [46,47], its metabolite AMPA [50] and phthalates [48].

### 4.3. Cells Treatment

**Figure 7:**
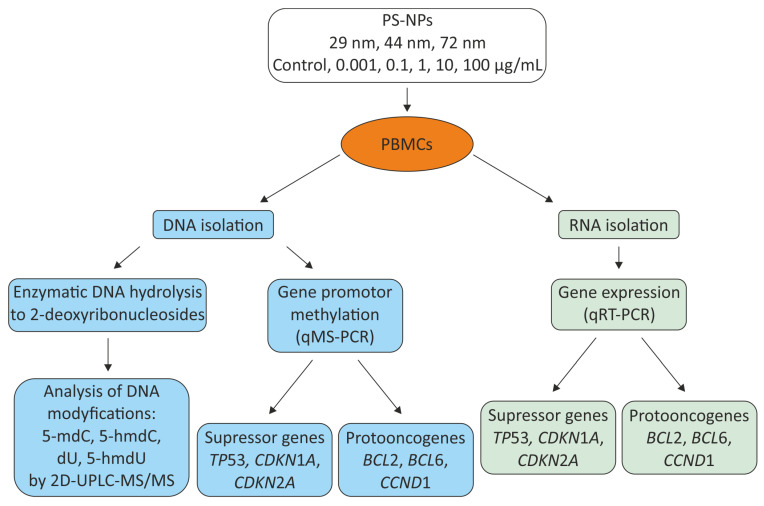
Flow chart of experimental design.

PS-NPs were dissolved in PBS with a pH of 7.4. The cytotoxicity of PS-NPs has been analyzed in our previous studies using the MTT assay [31] and flow cytometry with the fluorescent probes calcein-AM and propidium iodide [31,49]. Based on these findings, we selected concentrations for the current study ranging from 0.001 to 100 µg/mL. Within this range, PS-NPs exhibited no cytotoxicity. Cells were incubated with the nanoparticles for 24 h across 3 or 4 independent experiments involving four blood donors each. Throughout the incubation, the cells were suspended in RPMI supplemented with 10% FBS and penicillin/streptomycin solution (50 U/mL and 50 μg/mL, respectively), and maintained at 37 °C, 5% CO_2_. Following the incubation, the cells were centrifuged, the PS-NPs were discarded, and the cells were resuspended in PBS. Then, subsequent experiments were performed according to the flow chart (Figure 7).

### 4.4. Physico-Chemical Characterization of Non-Functionalized PS-NPs

The physical and chemical properties of tested non-functionalized NPs under investigation have been previously studied and described in our earlier publications [31,49]. Our findings revealed that PS-NPs tend to form local agglomerates due to strong interparticle and surface–particle interaction, although some particles also remained as individual entities [31]. The diameters of PS-NPs as specified by the manufacturer were consistent with those determined by our team using dynamic light scattering (DLS). Additionally, the ζ-potentials of PS-NPs in the RPMI medium were −41 ± 3 mV; −45 ± 2 mV and 56 ± 2 mV for NPs of 29 nm, 44 nm and 72 nm, respectively [31].

### 4.5. DNA Isolation and Enzymatic Hydrolysis to Deoxyribonucleosides

DNA isolation procedure was performed according to the protocol described previously by Guz et al. [80].

### 4.6. Determination of Epigenetic Modifications in DNA Isolated from Cells Exposed to PS-NPs

The analyses were performed using a method described by [81,82], with certain adjustments. These modifications primarily pertained to the adoption of mass spectrometry for 5-mdC determinations, replacing the previous UV detection method. Transition patterns that were selected as quantitative (242.2 > 126 and 254.2 > 133) for 5-methyl-2′-deoxycytydine and [^13^C_10_,^15^N_2_]-5methyl-2′-deoxycytydine, respectively) were acquired using MassLynx 4.2 software from Waters.

### 4.7. Methylation at Promoter Regions of Tumor Suppressor Genes (TP53, CDKN2A and CDKN1A) and Proto-Oncogenes (CCND1, BCL2, BCL6)

Genomic DNA from PBMC was isolated using PureLink™ Genomic DNA mini kit (Thermo Fisher Scientific, Waltham, MA, USA). Methylation levels in the promoter regions of *TP53*, *CDKN1A*, *CDKN2A*, *BCL2*, *BCL6* and *CCND1* genes were analyzed using quantitative methylation-specific real-time PCR (qMS-PCR). DNA sequence of promoter regions (from −500 to +100 bp) were obtained from the EPD (The Eukaryotic Promoter Database, https://epd.expasy.org/epd/ accessed on 1 January 2023) and used to design the primers by Methyl Primer Express™ Software v1.0 (Applied Biosystems, Waltham, MA, USA).

Isolated genomic DNA, CpG methylated Jurkat genomic DNA (positive control, fully methylated), 5-Azadc-treated Jurkat genomic DNA (negative control) (NEB, Ipswich, MA, USA) were converted using a sodium bisulfite kit. Chemical modification of 500 ng of genomic DNA and standards was performed by MethylCode™ Bisulfite Conversation kit (Thermo Fisher Scientific, Waltham, MA, USA). After sodium bisulfite conversion, the percentage of methylation index (MI) was assessed by qPCR with two pairs of primers for the methylated and unmethylated promoter region of the studied genes with FastStart Essential DNA Green Master (Roche, Basel, Switzerland) in the LightCycler^®^ 96 (Roche, Basel, Switzerland). The MI, expressed as a percentage of gene methylation (MI—%), was calculated for each sample using the following formula:

$$MI=\frac{1}{1+2(CtU-CtM)}\times 100\%,$$

where CtM and CtU are derived from qMSP with primers for the methylated and unmethylated gene sequences, respectively. Oligonucleotide sequences for the primers are presented in Supplementary Table S1.

### 4.8. Gene Expression

RNA extraction was performed using the TRIzol™ Plus RNA purification kit (Thermo Fischer Scientific, Waltham, MA, USA). The quality and quantity of all RNA samples were determined spectrophotometrically by a Multiskan GO multi-plate reader (Thermo-Fisher, Waltham, MA, USA). cDNA was synthesized from 100 ng of RNA, using Transcriptor First Strand cDNA Synthesis Kit (Roche, Basel Switzerland). Gene expression analysis was conducted by real-time PCR using FastStart SYBR Green Master (Roche, Basel, Switzerland).

Gene expression was normalized to the mean expression of all three housekeeping genes (*GAPDH*, *RPL13*, *RPLP0*) and was presented as normalized relative mRNA expression presented as log MNE (Mean Normalized Expression). MNE allows for sample comparison by normalizing expression with the geometric mention of the three reference genes (*GAPDH*, *RPL13*, *RPLP0*) using the following formula:

$$logMNE=\log_{10}\left(\;\frac{2^{Ct(ref)}}{2^{Ct(target)}}\right)\times 100,000$$

where Ct(ref) represents the geometric mean of three reference genes and Ct(target) is the cycle threshold of the target gene.

Oligonucleotide sequences for primers were designed using Primer-BLAST NCBI—NIH website: https://www.ncbi.nlm.nih.gov/tools/primer-blast/ accessed on 1 January 2023, based on DNA sequences from the NCBI Reference Sequences database: https://www.ncbi.nlm.nih.gov/pubmed/ accessed on on 1 January 2023. Oligonucleotide sequences for the primers are presented in Supplementary Table S2.

### 4.9. Statistical Analysis

Differences in the gene expression and methylation levels among PBMCs exposed to various concentrations of PS-NPs with diameters of 29 nm, 44 nm and 72 nm were assessed using analysis of variance (depending on the distribution of variables, either the one-way ANOVA test or the Kruskal–Wallis test was used). In multiple (two-sided) comparisons, differences were analyzed using an appropriate post hoc test (either Tukey’s test or Dunn’s test). The normality of distribution was evaluated using the Shapiro–Wilk test, while the assumption of homogeneity of variance was verified using Levene’s and Brown–Forsythe tests. The level of statistical significance was set at *p* < 0.05.

## 5. Conclusions

In this pioneering study, we examined the putative effect of non-functionalized PS-NPs to induce epigenetic alterations in human cells. Our findings reveal minor epigenetic perturbations in response to PS-NP exposure. Specifically, we observed no significant changes in global DNA methylation levels or methylation patterns within the CpG islands of key tumor suppressor genes (*TP53*, *CDKN2A*, and *CDKN1A*) and proto-oncogenes (*CCND1*, *BCl2*, *BCL6*) in peripheral blood mononuclear cells (PBMCs) following exposure to PS-NPs of varying diameters (29 nm, 44 nm, and 72 nm). Notably, an exception to this trend was the observed increase in *TP53* expression after incubation with PS-NPs of 29 nm and 44 nm diameters at the highest concentrations, along with elevated *CDKN2A* expression following exposure to 29 nm PS-NPs. Also, the level of 5-hmdU was increased at the highest concentrations of larger PS-NPs. These findings suggest a limited epigenetic impact of PS-NPs on the analyzed parameters in human PBMCs. Furthermore, our results indicate a size-dependent effect of PS-NPs, highlighting the importance of nanoparticle size in dictating their biological effects. In conclusion, while our study elucidates the limited epigenetic effect of PS-NPs on human PBMCs under the examined conditions, further research is warranted to comprehensively evaluate the safety and potential risks associated with nanoparticle exposure.

## Data Availability

The datasets presented during the current study are available from the corresponding author upon reasonable request.

## Supplementary Materials

The following supporting information can be downloaded at: https://www.mdpi.com/article/10.3390/ijms252312786/s1.
