# Supplementary material for: Impact of Short-Term Exposure to Non-Functionalized Polystyrene Nanoparticles on DNA Methylation and Gene Expression in Human Peripheral Blood Mononuclear Cells
Source: Int J Mol Sci. 2024 Nov 28;25(23):12786. doi: 10.3390/ijms252312786 (PMC11641298; doi:10.3390/ijms252312786)
Supplement: Supplementary file 1 [file ijms-25-12786-s001.zip › ijms-3324323-supplementary.pdf]

International Journal of *Molecular Sciences*

MDPI

Article

Impact of Short-Term Exposure to Non-Functionalized Polystyrene Nanoparticles on DNA Methylation and Gene Expression in Human Peripheral Blood Mononuclear Cells

Kinga Malinowska [1], Kateryna Tarhonska [2], Marek Foksiński [3], Paulina Sicińska [1], Ewa Jabłońska [2], Edyta Reszka [1], Ewelina Zarakowska [3], Daniel Gackowski [3], Karolina Górecka [4,5], Aneta Balcerczyk [6] and Bożena Bukowska [1,*]

[1] Department of Biophysics of Environmental Pollution, Faculty of Biology and Environmental Protection, University of Lodz, Pomorska Str. 141/143, 90-236 Lodz, Poland; kinga.malinowska@edu.uni.lodz.pl (K.M.); paulina.sicinska@biol.uni.lodz.pl (P.S.); edyta.reszka@biol.uni.lodz.pl (E.R.)

[2] Department of Translational Research, Nofer Institute of Occupational Medicine, Teresy Str. 8, 91-348 Lodz, Poland; kateryna.tarhonska@imp.lodz.pl (K.T.); ewa.jablonska@imp.lodz.pl (E.J.)

[3] Department of Clinical Biochemistry, Faculty of Pharmacy, Collegium Medicum in Bydgoszcz, Nicolaus Copernicus University in Toruń, 85-092 Bydgoszcz, Poland; marekf@cm.umk.pl (M.F.); ewelinaz@cm.umk.pl (E.Z.); danielg@cm.umk.pl (D.G.)

[4] The Bio-Med-Chem Doctoral School, University of Lodz, 90-237 Lodz, Poland; karolina.gorecka@edu.uni.lodz.pl

[5] Lodz Institutes of the Polish Academy of Sciences, University of Lodz, 90-237 Lodz, Poland

[6] Department of Oncobiology and Epigenetics, Faculty of Biology and Environmental Protection, University of Lodz, Pomorska 141/143, 90-236 Lodz, Poland; aneta.balcerczyk@biol.uni.lodz.pl

* Correspondence: bozena.bukowska@biol.uni.lodz.pl

Table S1. Primers for Methylation analyses

Gen	Forward Methylated DNA (MF)	Reverse Methylated DNA (MR)	Forward Unmethylated DNA (UF)	Reverse Unmethylated DNA (UR)
TP53 (P53)	CAACGATTTTCCCGAACTA	GGGTTGGGAGTGTGTTTTTTAT	GGGTTGGGAGTGTGTTTTTTAT	CAACAATTTTCCCAAACTAAAA
CDKN1A (P21)	TACGCGAGGTTTCGGGATCG	AAAAACGACCCGCGCTCG	TATGTGAGGTTTTGGGATTGG	AAAAACAACCCACACTCAACC
CDKN2A (P16)	TTCGAGTATTCGTTTACGGC	TTCTTCCTCCGATACTAACGA	TTTTTTGAGTATTTGTTTATGGT	CTTTCTTCCTCCAATACTAACAAA
CCND1	AGTTTTTAGAGGGTTGTCGGC	CTCTCGCTTCTACTACCCCG	GGAGTTTTTAGAGGGTTGTTGGT	CTCTCACTTCTACTACCCCAC
BCL2	TTAATTCGGGTTAGGGAGC	CGACCGATTTCCTATACGTA	TTTTTAATTTGGGTTAGGGAGT	ACCCAACCAATTTCCTATACATA
BCL6	GTTTTGTTATAGCGAAGGCGTC	AAAATTCCGATTCGAAACTCG	GAGTTTTGTTATAGTGAAGGTGTT	AAAATTCCAATTCAAAACTCAAA

Table S2. Primers for expression analyses

**Gene**	**Forward Primer 5’-3’**	**Reverse Primer 5’-3’**
TP53 (p53)	GCTCAAGACTGGCGCTAAAA	GTCACCGTCGTGGAAAGC
CDKN1A (p21)	TGTCTTGTACCCTTGTGCCTC	TGGTAGAAATCTGTCATGCTGGT
CDKN2A (p16)	CACATTCATGTGGGCATTTC	TGCTTGTCATGAAGTCGACAG
CCND1	GAGGAGCTGCTGCAAATGGA	GGAGGGCGGATTGGAAATGA
BCL2	AGTACCTGAACCGGCACCT	GCCGTACAGTTCCACAAAGG
BCL6	TCAGATTCTAGCTGTGAGAACGGG	GGCAGCGGTCACACTTGTAG
GAPDH	AGCCACATCGCTCAGACAC	GCCCAATACGACCAAATCC
RPL0	TCTACAACCCTGAAGTGCTTGAT	CAATCTGCAGACAGACACTGG
RPL13	CAAGCGGATGAACACCAAC	TGTGGGGCAGCATACCTC
